# A Handy Endoscopic Tool: Modification of a Latex Glove for Successful Foreign Body Extraction

**DOI:** 10.7759/cureus.97138

**Published:** 2025-11-18

**Authors:** Ryan Burd, Min Kyung Kang, Samuel Aulick, Sandesh Karki, Ernesto Rodriguez, Rajesh Veluvolu, Indraneel Chakrabarty

**Affiliations:** 1 Internal Medicine, Southwest Healthcare Medical Education Consortium, Temecula, USA; 2 Clinical Sciences, West Virginia School of Osteopathic Medicine, Lewisburg, USA; 3 Gastroenterology, Southwest Healthcare Medical Education Consortium, Temecula, USA

**Keywords:** endoscopy, esophagogastroduodenoscopy, foreign body, latex hood, overtube, pylorus

## Abstract

While foreign body (FB) ingestion primarily affects children, it commonly occurs in adults during contextual meals. This case report presents a 39-year-old male who accidentally swallowed a piece of his dental bridge appliance while eating. Retrieval was faced with the challenge of an exposed metal edge visible during endoscopy, placing the patient at risk for mucosal laceration and perforation. A protective latex hood was created from a surgical glove in the absence of an available premanufactured hood or appropriately sized overtube. This case highlights a simple, resourceful technique for mucosal protection during high-risk FB extraction when standard equipment is unavailable.

## Introduction

Foreign body (FB) ingestion is defined as the intentional or unintentional swallowing of FBs, with an annual incidence of approximately 120,000 cases in the United States [1]. FB ingestion most commonly occurs in the context of eating and is primarily a pediatric concern with only 25% of cases affecting adults [2]. In the following case report, we present a 39-year-old male with no past medical history who unintentionally ingested a piece of his dental bridge appliance while eating. The patient immediately presented to the emergency department after ingestion where removal of the FB occurred without complications using esophagogastroduodenoscopy (EGD). During endoscopic removal, it was found that the patient’s dental bridge contained an exposed metal edge that was likely to irritate and possibly lacerate or perforate the esophageal mucosa. Premanufactured hoods and appropriately sized overtubes are attachable endoscopic tools that serve to protect the esophageal and gastric mucosa to prevent injury or complications. In the absence of such devices, innovation and creativity were required to fashion a makeshift latex hood using a surgical glove. Lack of appropriate equipment can occur in any endoscopic suite, however this may occur more frequently in rural and community medical centers. Such innovation is significantly underepresented in present medical literature and this report is among the few globally documented cases to meet the challenge of gastrointestinal ingenuity that is necessary for safe FB removal in the absence of traditional protective equipment.

## Case presentation

A 39-year-old male with no past medical history presented to the emergency department immediately after ingesting a dental bridge while eating a hamburger, with residual mild abdominal discomfort. The patient was not taking any medications or supplements at home. The patient further denied any past surgical or family history, and social history was significant for methamphetamine smoking, with the last use five years prior to presentation. 

**Figure 1:**
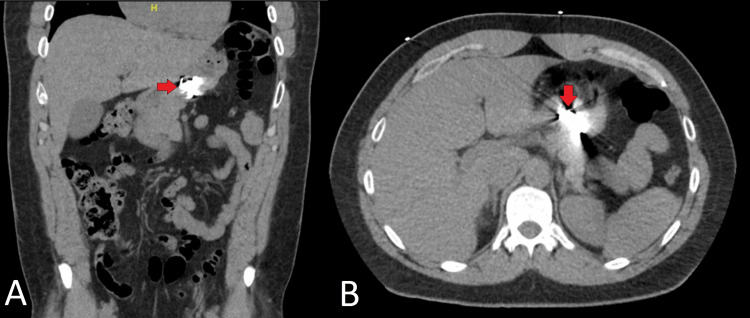
Computed tomography scan of foreign body ingestion (see arrow) Coronal (A) and axial (B) views of the abdomen and pelvis revealing a high-density foreign object in the gastric body.

**Figure 2:**
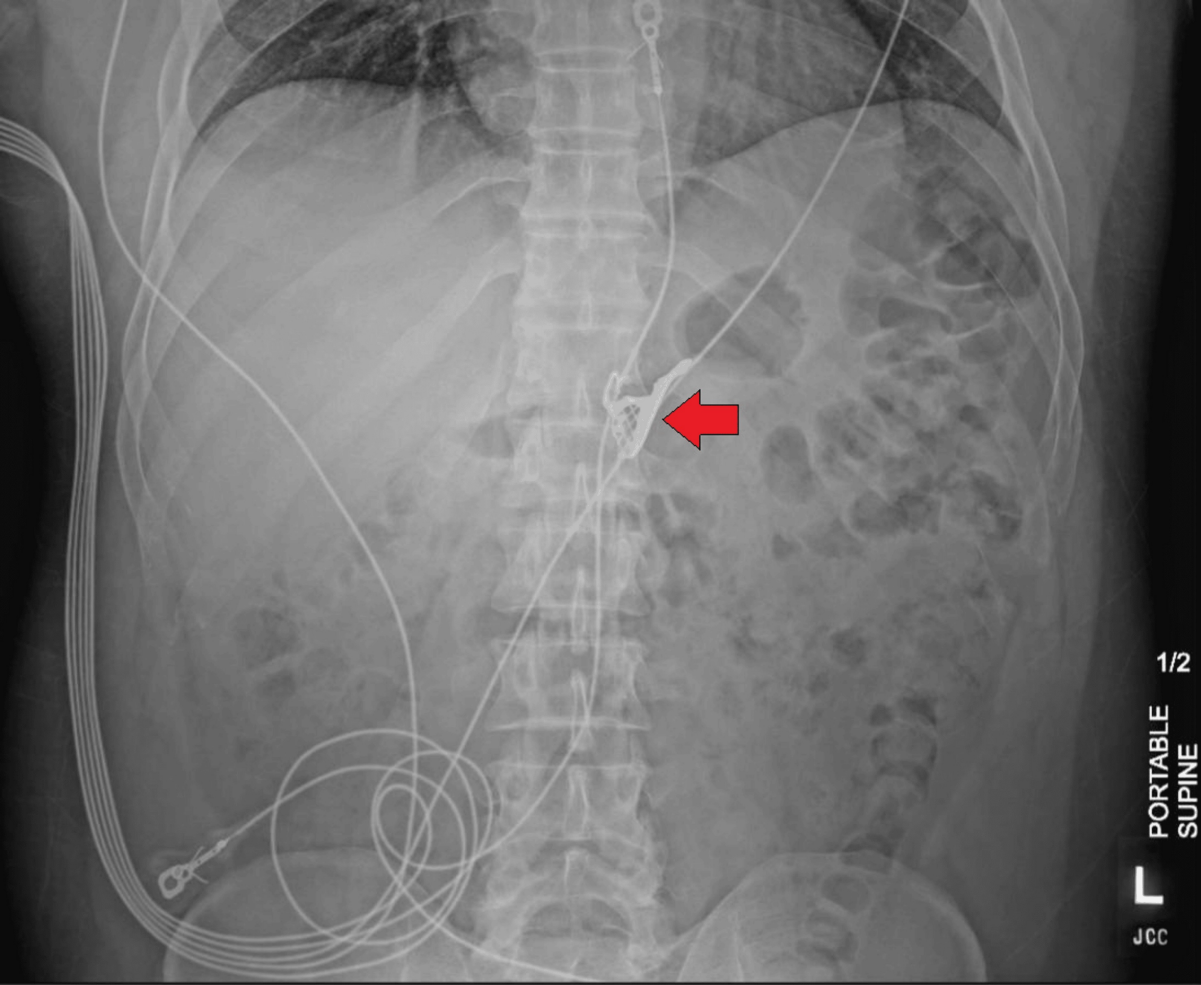
Abdominal radiograph of the foreign body (see arrow) Abdominal radiograph revealing retention of foreign body in the gastric body without distal migration.

Vitals upon admission were significant for blood pressure of 143/88mmHg. Initial complete blood count and complete metabolic panel were unremarkable. Urine toxicology was significant for cannabinoids. A computed tomography (CT) of the abdomen and pelvis revealed a high-density foreign object in the gastric body without evidence of obstruction (Figure 1). The patient was started on a proton pump inhibitor (PPI) and serial abdominal radiographs demonstrated no migration of the FB in the gastric body (Figure 2). The gastroenterology service was consulted for planned endoscopic FB removal.

**Figure 3:**
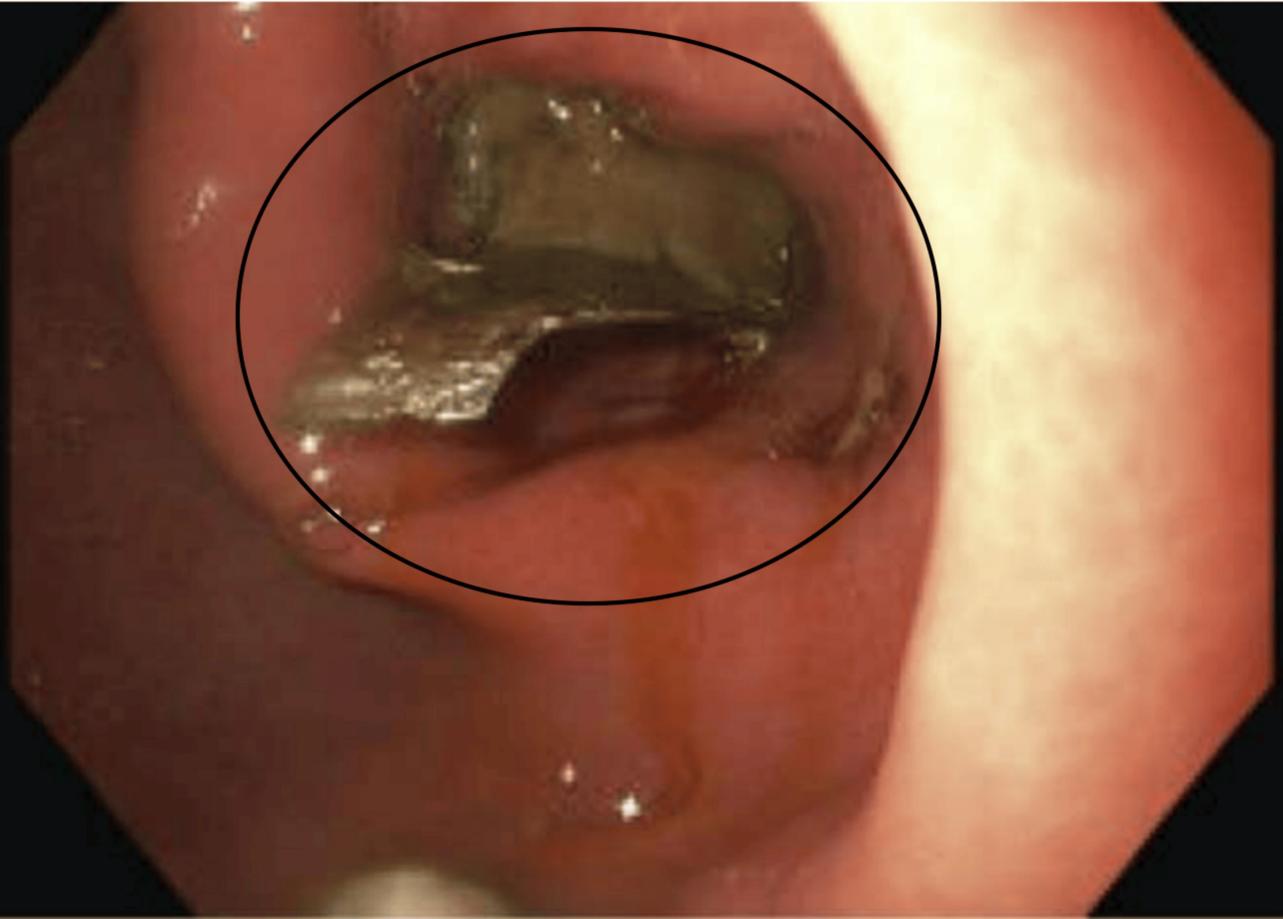
Endoscopic view of foreign body ingestion (see oval) Endoscopic view of the foreign body stuck in the pylorus with the sharp edge evident within the oval.

**Figure 4:**
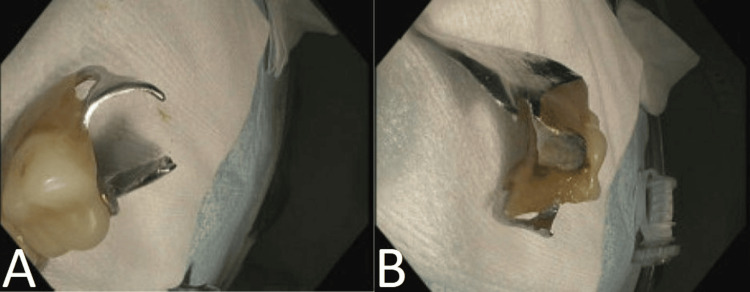
Endoscopic view of foreign body post-retrieval Front (A) and lateral (B) views of ingested dental bridge post-retrieval.

**Figure 5:**
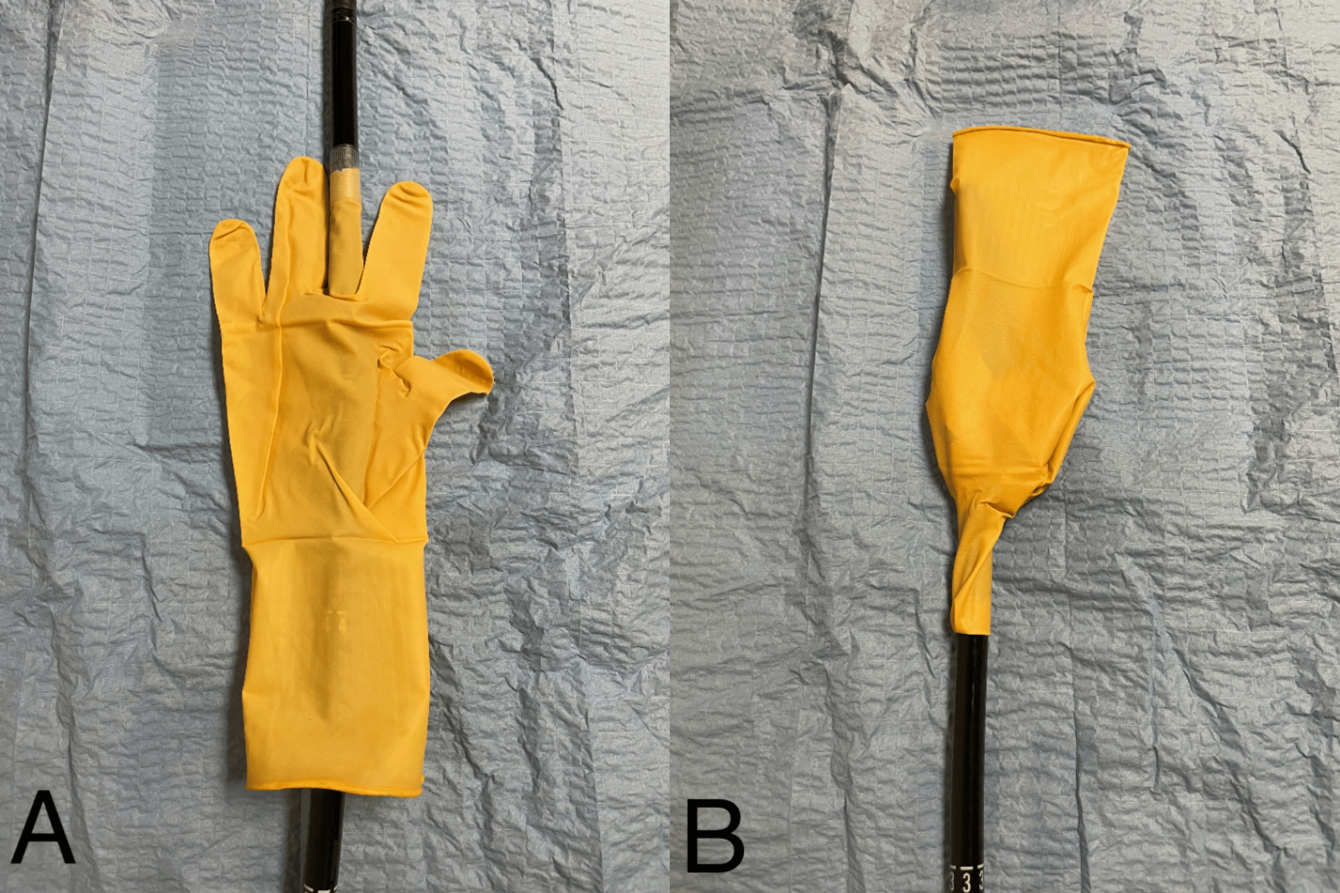
Enodscopic latex hood constructed from modified size 8 surgical glove Protective latex hood constructed from a size 8 surgical glove modified about an esophagogastroduodenoscopy endoscope with demonstrated orientation during advancement (A) or pushing the endoscope distally, and retraction (B) or pulling the endoscope proximally.

During EGD, a single dental bridge was found impacted in the pylorus (Figure 3), which was immediately disimpacted. Visualization of the dental bridge revealed exposed sharp metal ends (Figure 4) with concern for laceration or perforation along the gastric and esophageal lumen if removed without additional protection. A premanufactured hood or appropriately sized overtube was not available at the time of the procedure, so a makeshift latex hood (Figure 5) was created with a size eight sterile glove. The tip of the glove's third digit was removed, placed approximately 10cm over the endoscope and secured with adhesive tape to complete safe and successful retrieval. The patient was subsequently discharged with a 30-day prescription of pantoprazole 40mg daily to reduce residual gastric irritation.

## Discussion

FB ingestion is a common gastrointestinal complaint with an incidence of approximately 120,000 cases in the United States annually [1], primarily affecting children under six, representing approximately 75% of cases [2]. Among FB ingestions globally, objects are typically ingested under the context of eating, most commonly fish bones, shells and other non-metabolizable components of food [3-6]. Among adults, FB ingestion is also commonly experienced with digestive diseases, alcohol use, drug use and psychiatric disorders [5]. Modern retrospective analyses indicate that up to 25% of FB ingestions are intentional and common complications include hemorrhage and gastrointestinal perforation [5,7]. Reported risk factors for associated complications include age ≥60, presence of the FB ≥24h and esophageal location [7]. While the unintentional FB ingestion in this case presentation occurred during mealtime, it uniquely deviates from associated statistics based on the ingested FB, localization within the gastric body and successful retrieval that occurred without complications.

Current guidelines regarding management of ingested FBs recommend the use of flexible endoscopy for FBs located distal to the upper esophageal sphincter and retrieval devices reportedly depend on the size and characteristics of the FB [8]. Certain FBs with elevated risk for complications upon removal may benefit from the use of an overtube, which houses FBs during extraction and protects the esophageal mucosa [8]. Overtube use for FB extraction was first reported by Witzel et al. (1974) with use demonstrating superior protection in subsequent trials for FB ingestion and food bolus impaction of the esophagus [9,10]. An alternative means of protection was proposed by Bertoni et al. (1996) in the form of a latex hood attached around an endoscope in an inverted fashion and flipped during retraction through the lower esophageal sphincter [11]. Such devices continue to be used alongside or in place of overtubes for FB extraction and choice depends on operator preference and experience.

Sporadic global case reports have described the use of alternative objects functioning as makeshift hoods in the absence of traditional equipment during endoscopic extraction of FBs. International reports have described the attachment of rubber and plastic objects including gloves, condoms and transfusion pressure infusors to the end of an endoscope to protect the esophageal mucosa in the absence of a formal hood or overtube [12-14]. A case report by Kao et al. (2000) describes the use of a latex glove cuff to assist with FB extraction in a similar manner and was previously the only report of its kind in the United States [15].

As such, this case report is among the first to report successful FB extraction with a makeshift latex hood, globally and nationally. It uniquely utilized an entire latex glove fashioned proximal to the distal end of the endoscope to avoid unnecessary cutting or sizing of the hood, which minimized the patient's time under anesthesia. While it has been demonstrated that alternatives to hoods and overtubes can be feasible when such devices are unavailable, this report highlights the importance of proper equipment for both the extraction of unique FBs and protection of local structures. Limitations of using improvised equipment include suboptimal visibility, mobility and a lack of industry standardization.

## Conclusions

Given that FB ingestion is common, current guideline-directed management of FB removal is especially important in light of potential complications including laceration or perforation of the esophageal mucosa during extraction. In this case presentation, no overtube or premanufactured hood was available during endoscopy for safe extraction of an ingested dental bridge, and a makeshift hood was created from a surgical glove, completing the procedure without further complications. This case is an important contribution to the medical literature because safe endoscopic extraction of FBs without access to mucosa protective equipment remains a global challenge, particularly among rural and community medical centers.
